# Case report: A novel reciprocal ROS1-CD74 fusion in a NSCLC patient partially benefited from sequential tyrosine kinase inhibitors treatment

**DOI:** 10.3389/fonc.2022.1021342

**Published:** 2022-10-31

**Authors:** Xugang Zhang, Baoming Wang, Chunyang Wang, Chengde Liao, Shiping Wang, Ran Cao, Tonghui Ma, Kun Wang

**Affiliations:** ^1^ The Affiliated Anning First People’s Hospital, Kunming University of Science and Technology, Kunming, Anning First People’s Hospital, Yunnan, China; ^2^ Department of Translational Medicine, Genetron Health (Beijing) Co. Ltd., Hangzhou, China; ^3^ Department of Radiology, The Third Affiliated Hospital of Kunming Medical University, Yunnan Cancer Hospital, Yunnan, China

**Keywords:** ROS1, sequencing, inhibitor, sequential detection, NGS

## Abstract

**Background:**

The clinical significance of majority oncogenic novel fusions is still unknown due to scarcity. Reciprocal *ROS1* translocation is a rare form of *ROS1* fusion and has not yet been clearly analyzed.

**Case presentation:**

A 44-year-old Chinese woman with a large dimension in the left lobe of the lung was admitted to the hospital with IVB lung adenocarcinoma. It was discovered that intron 28 of *ROS1* and intron 6 of *CD74* produced a unique reciprocal *ROS1* rearrangement. In addition, the dual *CD74-ROS1* fusions were discovered using the RNA next-generation sequencing (NGS) findings. Although benefiting from crizotinib and lorlatinib sequential treatment, the overall prognosis of the patient was relatively poor, whose progression-free survival was 4 and 5 months for crizotinib treatment and lorlatinib treatment, respectively.

**Conclusion:**

In summary, a novel *ROS1-CD74* fusion identified by DNA NGS was translated into dual *CD74-ROS1* transcripts. Furthermore, this patient with non–small cell lung cancer benefited from consecutive tyrosine kinase inhibitor therapy. Our discovery broadened the range of targetable ROS1 fusions and underlined the importance of sequential DNA and RNA sequencing in identifying uncommon but beneficial fusions, which eventually bring benefits to the patients.

## Introduction

Approximately 2% of East Asian patients with non–small cell lung cancer (NSCLC) have *ROS1* rearrangement, which has defined a unique molecular subtype (1). The U.S. Food and Drug Administration had authorized a number of targetable medications that target this kind of aberration. For instance, crizotinib, lorlatinib, and entrectinib are granted in patients with NSCLC carrying *ROS1* fusions (2–4). The clinical application of these targeted drugs offers a significant improvement in both survival and quality of life of patients with cancer. With the development of precision medicine, DNA NGS is quickly replacing fluorescence *in situ* hybridization (FISH) and Immunohistochemistry (IHC) as the primary method for identifying oncogenic fusions and may offer more detailed information. More than 40 *ROS1* partners have been found by NGS thus far (5).

Despite the individuals with *ROS1* rearrangements responding dramatically to crizotinib therapy, a prior study showed that the response time differs among patients with different clinical and genetic features (6). To choose the appropriate therapeutic approaches, it is crucial to recognize druggable ROS1 fusions. Previous research rarely reported single reciprocal ROS1 fusions, which kept ROS1 as the 5′ fusion gene without their counterpart of the 3′ ROS1 fusion variant (7). Although it occurs very rarely, research on this uncommon ROS1 fusion is vital to broaden the genetic landscape of usable ROS1 fusions and to provide personalized precision medicine.

In this study, for the first time, a non-smoker female patient with NSCLC with a novel reciprocal ROS1-CD74 fusion without tyrosine kinase domain was discovered in the DNA NGS, which could be transcribed into dual CD74-ROS1 fusions at transcription level. Furthermore, the patient benefited from sequential crizotinib and lorlatinib therapy.

## Case presentation

**Figure 1:**
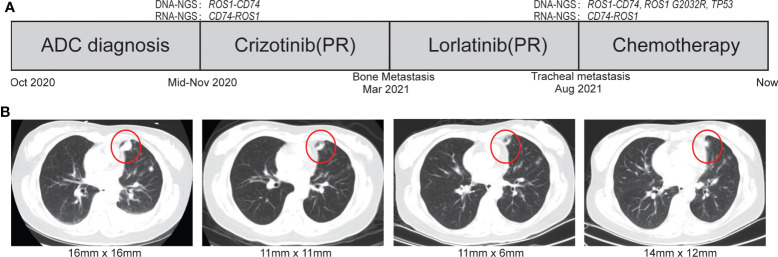
The treatment scheme and representative CT scan images during treatment. **(A)** Treatment scheme. **(B)** The representative CT scan of during treatment courses. ADC, adenocarcinoma; SD, stable disease; PR, partial response.

This case report was approved by the Ethics Committee of the Anning First People’s Hospital 2022-008(others)-01. A 44-year-old Chinese female patient was admitted to the hospital with paroxysmal abdominal pain in October 2020. Chest computed tomography (CT) revealed the shadow of a mass in the left lobe of lung. IVB lung adenocarcinoma was diagnosed on the basis of the combination of pathological results of biopsy specimen and CT (**Figures 1A, B**).

**Figure 2:**
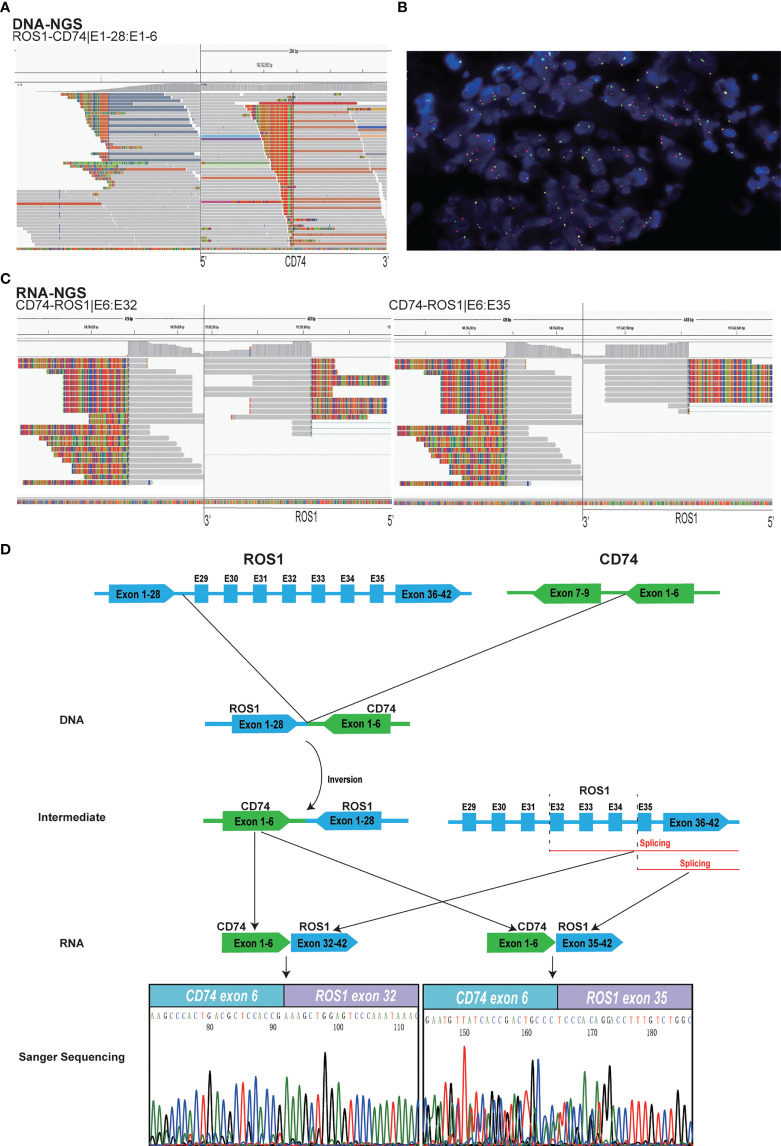
Identification of a novel ROS1 fusion. **(A)** DNA NGS analysis of the genomic ROS1-CD74 novel fusion. **(B)** FISH analysis showed fused yellow signals (negative signal), single green signals (positive signal), and single red signals (positive signal) in the patient’s specimen. **(C)** RNA NGS analysis of the dual CD74-ROS1 fusions at transcript level. **(D)** Possible schematic diagram of ROS1-CD74 fusion detected by DNA-based NGS, but CD74-ROS1 fusions were identified by RNA-based NGS. NGS, next-generation sequencing; TKI, tyrosine kinase inhibitor; FISH, fluorescence *in situ* hybridization.

To explore the therapeutic opportunities, a comprehensive molecular profiling of tumor biopsy specimen from the lung was submitted for NGS with an 825 cancer-related gene DNA panel (Onco Panscan™) at Genetron Health, Inc. (Beijing, China). A novel reciprocal *ROS1* rearrangement generated by intron 28 of *ROS1* and intron 6 of *CD74* was identified by sequencing (**Figure 2A**). At the same time, three gene alterations co-existent with the novel *ROS1* fusion were also detected in primary tumor tissue samples. They were *SETD2* p.I1476Lfs*7 (variant allele frequency (VAF), 20.08%), *TP63* p.H247R (variant allele frequency (VAF), 19.49%), and *ACVR2A* p.V433del (variant allele frequency (VAF), 16.38%). The gene fusion was confirmed by FISH with *ROS1* break-apart probe (**Figure 2B**). Interestingly, dual functional fusions of *CD74-ROS1* (E6:E32) and *CD74-ROS1* (E6:E35) were presented at the transcriptional level by targeted RNA sequencing (Fusioncapture™, Genetron Health) (**Figure 2C**), which was confirmed by Sanger’s sequencing (**Figure 2D**).

Crizotinib was firstly administrated on the basis of the positive *ROS1* fusion result by sequencing. Compared with the naive period, CT scan revealed a significant decrease in lung focus after 2 months treatment (16 × 16 m to 11 × 11 mm). However, the patient developed resistance to crizotinib after 4 months reflected by bone metastasis. Because of her own physical condition and other reasons, the patient did not take a puncture or liquid biopsy sample for genetic testing to determine the mechanism of resistance of crizotinib. At the patient’s request, the patient immediately voluntarily received the third-generation *ROS1* tyrosine kinase inhibitor (TKI) lorlatinib treatment. The great treatment outcome was reflected by the significant decrement of the tumor size (16 × 16 mm to 11 × 6 mm). Unfortunately, the lorlatinib treatment had to be discontinued due to the trachea metastasis after 5 months therapy (**Figures 1A, B**). DNA NGS was performed on the metastasis tumor specimen after resistance to the lorlatinib, and the novel reciprocal *ROS1-CD74* fusion was detected again, which was also confirmed by FISH result (**Figures 1A**, **2B**). In addition, except the three gene variations in the primary tissue of the tumor that were still detected, two other mutations, *TP53* p.R248W (12.3%) and *ROS1* p.G2032R (4.5%), were newly discovered in the puncture samples after lorlatinib resistance. Among which, *ROS1* G2032R has been reported to be a potential drug resistance site of crizotinib and lorlatinib. The patient was subsequently treated with chemotherapy but sadly died of tumor progression in May 2022.

Here, a novel reciprocal *ROS1-CD74* fusion identified by DNA NGS, which transcribed into dual *CD74-ROS1* fusions (E6:E32; E6:E35) at the RNA level, showed robust but unsustainable response to sequential crizotinib and lorlatinib administration. To our knowledge, this is the first report of a reciprocal *ROS1-CD74* fusion that did not have an intact kinase domain in DNA NGS that could benefit from *ROS1* inhibitor; the mechanism of the drug response to this rare fusion has also been elucidated.

It is frequently believed that the presence of an intact kinase domain is necessary for kinase inhibitors’ treatment identified by DNA sequencing. Reciprocal fusions with incomplete kinase domain were occasionally seen alone in patients with NSCLC and usually considered as non-functional (8). Xu et al. reported that a patient with lung adenocarcinoma carried a *ROS1-ADGRG6* fusion at the DNA level and responded to crizotinib (9). However, this fusion was generated after *EGFR*-TKI resistance and had not been verified by RNA NGS; the mechanism of drug response also had not been studied. For the first time, we described the detection of a previously unreported reciprocal fusion of *ROS1-CD74* as a driver event in a patient with lung cancer. In this patient, exons 1–28 of *ROS1* were fused to exons 1–6 of *CD74* in the opposite direction at the chromosomal level. The raw data were queried, and none of the reads supporting the classical form of *CD74-ROS1* fusion was confirmed. Interestingly, this reciprocal form fusion could be transcribed into rare form of dual *CD74-ROS1* (E6:E32; E6:E35) fusions. Our result proved that the novel reciprocal fusion may be functional and should not be ignored during DNA NGS detection. This study also emphasized that sequential DNA and RNA sequencing could provide more precise evidence and optimize the targeted therapy strategy.

Although different *ROS1* fusion partners have been already identified, few data are currently addressed on how different fusion partners affect the efficacy of *ROS1*-TKIs. A previous study proved that the patients carried single non–*CD74-ROS1* fusions could get better prognostic outcomes than those with *CD74-ROS1* fusions during crizotinib treatment (6), whereas the studies on the efficacy of *ROS1*-TKI in patients with two *ROS1* fusions are rare and the conclusions are conflicting. Lan et al. identified a patient with NSCLC with non-reciprocal/reciprocal *ROS1* rearrangement (*ROS1-FBXL17* fusion co-existing with *CD74-ROS1* fusion), who obtained an excellent response to crizotinib, and the progression-free survival (PFS) was up to 17 months (10). However, in another study, a patient with lung adenocarcinoma harboring complex *ROS1* fusions (*GK-ROS1* and *SDC4-ROS1*) showed resistance only 8 months after crizotinib therapy (7). These inconsistent findings suggest that further investigations are needed to explore the efficacy of *ROS1* inhibitors in dual *ROS1* fusion. Furthermore, the reciprocal genomic ROS1-CD74 fusion was reported before in the NSCLC; however, whether it could transcribe into functional transcript and be targeted by TKI was unknown (11). In this case, we found a patient with NSCLC with novel dual reciprocal *ROS1* fusions (*CD74-ROS1*, E6:E32; *CD74-ROS1*, E6:E35), which is different from the non-reciprocal/reciprocal form of double *ROS1* fusions reported in other reports. The patient presented observably response to different *ROS1*-TKIs, but the effective duration was significantly shorter than the median PFS in patients with NSCLC (crizotinib, 4 months *vs*. 12.6 months; and lorlatinib, 5 months *vs*. 8.5 months, respectively). The reason of inferior response in our case may partially attribute to the tumor heterogeneity. A previous study demonstrated that a single tumor cell could only contain one RNA fusion isoform and that patients with multiple isoforms had worse prognostic outcomes (12). The co-existence of *CD74-ROS1* (E6:E32) and *CD74-ROS1* (E6:E35) fusion in the patient represented the complicated tumor heterogeneity, which may acted as a poor predictive marker with *ROS1* translocations similar to non-reciprocal/reciprocal *ALK* translocation in patients with NSCLC (13).

In addition, the clinical significance of *SETD2* p.I1476Lfs*7 that co-mutated with *CD74-ROS1* translocations in this patient was unclear. Previous studies have shown that concomitant mutations can also lead to relatively poor response to crizotinib in patients carrying *CD74-ROS1* translocations. This may be one of the other reasons why our patient was relatively ineffective in receiving *ROS1*-TKI treatment. The efficacy of *ROS1* inhibitors in patients with dual *ROS1* fusions should be explored in the future.

Several limitations need to discuss. First, the tumor specimen after resistance to crizotinib was unavailable; therefore, the occurrence time of *ROS1* G2032R mutation is undetermined. In addition, although the concomitant mutations such as *TP53* R248W were detected in samples of patients resistant to loratinib treatment, the reason for the loratinib resistant mechanism is unknown. Given the limitation of patient number, the clinical function of the dual *ROS1* fusions in cancer should be confirmed by further research.

In summary, a patient with NSCLC with novel reciprocal *ROS1-CD74* fusion, which could be transcribed into dual *CD74-ROS1* fusions was first identified and benefited from the sequential crizotinib and lorlatinib therapy. Detection of dual *CD74-ROS1* fusion may be associated with poor survival outcomes with *ROS1*-TKIs. Moreover, sequential DNA and RNA sequencing is essential to promote the fusion detection precision and to predict the benefit to targeted therapy.

## Data availability statement

The original contributions presented in the study are included in the article/supplementary material. Further inquiries can be directed to the corresponding authors.

## Ethics statement

The studies involving human participants were reviewed and approved by Anning First People’s Hospital. The patients/participants provided their written informed consent to participate in this study.

## Author contributions

XZ and BW contributed to the experiment performing and manuscript writing. CW and CL participated to the data analysis. SW and RC provided the clinical samples and relevant information. TM and KW designed and optimized the experiment. All authors contributed to the article and approved the submitted version.

## Funding

Kunming Municipal Health Committee Health Research Project (2020-04-02-115).

## Conflict of interest

Authors BW, CW and TM are employed by Genetron Health Beijing Co. Ltd.

The remaining authors declare that the research was conducted in the absence of any commercial or financial relationships that could be construed as a potential conflict of interest.

## Publisher’s note

All claims expressed in this article are solely those of the authors and do not necessarily represent those of their affiliated organizations, or those of the publisher, the editors and the reviewers. Any product that may be evaluated in this article, or claim that may be made by its manufacturer, is not guaranteed or endorsed by the publisher.
